# (*N*,*N*,*N*′,*N*′-Tetra­methyl­ethylenediamine-κ*N*)bis­(2,4,6-trimethyl­phenolato-κ*O*)germanium(II)

**DOI:** 10.1107/S160053681200503X

**Published:** 2012-02-17

**Authors:** Oleksii Brusylovets, Oleg Yrushnikov, Dina Naumova, Nikolai Klishin, Eduard Rusanov

**Affiliations:** a Department of Chemistry, Kiev National Taras Shevchenko University, Volodymyrska Street 64, 01601 Kiev, Ukraine; b Institute of Organic Chemistry, National Academy of Sciences of Ukraine, Chervonatkatska Street 60, 02660 Kiev, Ukraine

## Abstract

In the title compound, [Ge(C_9_H_11_O)_2_(C_6_H_16_N_2_)], the Ge^II^ atom is coordinated in a distorted trigonal–pyramidal geometry by two O atoms belonging to two 2,4,6-trimethyl­phenolate ligands and one N atom of a tetra­methyl­ethylenediamine ligand. Comparing the structure with published data of similar compounds shows that the Ge—O bonds are covalent and the Ge—N bond is coordinated.

## Related literature

For the synthesis and chemistry of aryl­oxygermylene–amine complexes, see: Bonnefille *et al.* (2006 ▶). For related compounds, see: Huang *et al.* (2009 ▶); Leung *et al.* (2007 ▶); Seigi & Hoffman (1996 ▶); Weinert *et al.* (2003 ▶); Wetherby *et al.* (2008 ▶); Zemlyansky *et al.* (2003 ▶).
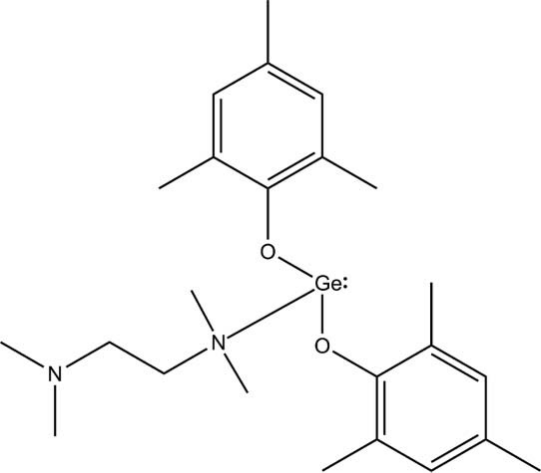


## Experimental

### 

#### Crystal data

[Ge(C_9_H_11_O)_2_(C_6_H_16_N_2_)]
*M* *_r_* = 459.15
Triclinic, 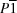

*a* = 10.9026 (3) Å
*b* = 11.5495 (3) Å
*c* = 12.4890 (3) Å
α = 92.552 (1)°
β = 113.853 (1)°
γ = 117.838 (1)°
*V* = 1217.73 (5) Å^3^
*Z* = 2
Mo *K*α radiation
μ = 1.28 mm^−1^
*T* = 173 K
0.50 × 0.35 × 0.29 mm

#### Data collection

Nonius KappaCCD diffractometer
Absorption correction: multi-scan (*DENZO*/*SCALEPACK*; Otwinowski & Minor, 1997 ▶) *T*_min_ = 0.567, *T*_max_ = 0.708
18801 measured reflections
5132 independent reflections
4480 reflections with *I* > 2σ(*I*)
*R*_int_ = 0.032

#### Refinement

*R*[*F* ^2^ > 2σ(*F* ^2^)] = 0.030
*wR*(*F* ^2^) = 0.082
*S* = 1.02
5132 reflections
262 parameters
H-atom parameters constrained
Δρ_max_ = 0.38 e Å^−3^
Δρ_min_ = −0.30 e Å^−3^

### 

Data collection: *COLLECT* (Nonius, 1998 ▶); cell refinement: *DENZO*/*SCALEPACK* (Otwinowski & Minor, 1997 ▶); data reduction: *DENZO*/*SCALEPACK*; program(s) used to solve structure: *SIR2004* (Burla *et al.*, 2005 ▶); program(s) used to refine structure: *SHELXL97* (Sheldrick, 2008 ▶); molecular graphics: *ORTEP-3* (Farrugia, 1997 ▶); software used to prepare material for publication: *SHELXL97*.

## Supplementary Material

Crystal structure: contains datablock(s) I, global. DOI: 10.1107/S160053681200503X/hy2510sup1.cif

Structure factors: contains datablock(s) I. DOI: 10.1107/S160053681200503X/hy2510Isup2.hkl

Additional supplementary materials:  crystallographic information; 3D view; checkCIF report

## Figures and Tables

**Table 1:** Selected bond lengths (Å)

Ge1—O1	1.8760 (13)
Ge1—O2	1.8674 (13)
Ge1—N1	2.1261 (16)

**Fig. 1.**
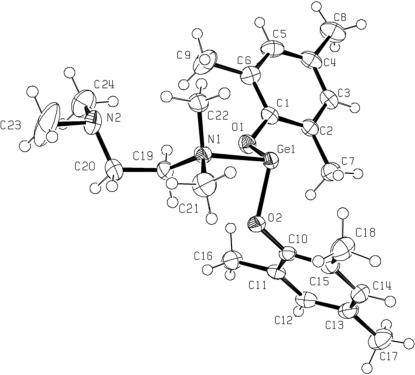
The molecular structure of the title compound, with displacement ellipsoids drawn at the 50% probability level.

## supplementary crystallographic information

### Comment

Three coordinated germanium compounds are rare enough, because this state is not typical. Bulky substituents have been used for stabilization of this state. Previously well-studied similar crystal structures, where germanium is surrounded by two oxygen atoms and one nitrogen atom, contain Ge(II) as a central atom. These compounds possess similar values of bonds lengths, bond angles, and also similar structures (Huang *et al.*, 2009; Leung *et al.*, 2007; Seigi & Hoffman, 1996; Wetherby *et al.*, 2008; Zemlyansky *et al.*, 2003). A dimeric crystal structure of this type, [Ge(2,4,6-Me_3_C_6_H_2_O)_2_]_2_, without use of a tertiary amine as a stabilizer was synthesized (Weinert *et al.*, 2003). Such compounds as (MesO)_2_Ge(NR_3_) [NR_3_ = Et_2_NH, (C_6_H_11_)_2_NH, Et_3_N, dabco, tmeda] (MesO = 2,4,6-trimethylphenolate; dabco = 1,4-diazabicyclo[2.2.2]octane; tmeda = N,N,N',N'-tetramethylethane-1,2-diamine] were also synthesized, but as a wax material and the authors argue that these substances can not be obtained in crystalline form (Bonnefille *et al.*, 2006). Studying the literature we have noticed that the length of a covalent bond Ge—O is in an interval 1.760–1.910 Å, the length of a coordinate bond Ge—O is in an interval 2.226–2.403 Å, the length of a covalent bond Ge—N lies in an interval 1.890–1.956 Å, and the length of a coordinate bond Ge—N lies in an interval 2.022–2.286 Å.

### Experimental

To a stirred solution of dichlorogermylene-dioxane (2.32 g, 9.98 mmol) in 30 ml of toluene was added 2,4,6-trimethylphenol (2.72 g, 19.96 mmol) in 20 ml of toluene and tetramethylethylenediamine (6.1 ml, 39.92 mmol). The mixture was stirred for 16 h at 200°C. Precipitate of the quaternary amine formed during the reaction was filtrated from this solution. The filtered solution was evaporated to a wax material. Obtained waxy material (0.8 g) was dissolved in 5 ml of diethylether and left at -25°C. Within three days transparent crystals dropped out of the solution and were filtrated off (yield: 0.72 g, 90%). Analysis, calculated for C_24_H_38_GeN_2_O_2_: C 62.79, H 8.28, N 6.10%; found: C 62.71, H 8.24, N 6.06%.

### Refinement

H atoms were positioned geometrically and refined as riding atoms, with C—H = 0.95 (CH), 0.99 (CH_2_) and 0.98 (CH_3_) Å and with *U*_iso_(H) = 1.2(1.5 for methyl)*U*_eq_(C).

### Crystal data

**Table:**

[Ge(C_9_H_11_O)_2_(C_6_H_16_N_2_)]	*Z* = 2
*M**_r_* = 459.15	*F*(000) = 488
Triclinic, *P*1	*D*_x_ = 1.252 Mg m^−^^3^
Hall symbol: -P 1	Mo *K*α radiation, λ = 0.71073 Å
*a* = 10.9026 (3) Å	Cell parameters from 9278 reflections
*b* = 11.5495 (3) Å	θ = 2.2–26.6°
*c* = 12.4890 (3) Å	µ = 1.28 mm^−^^1^
α = 92.552 (1)°	*T* = 173 K
β = 113.853 (1)°	Block, colourless
γ = 117.838 (1)°	0.50 × 0.35 × 0.29 mm
*V* = 1217.73 (5) Å^3^	

### Data collection

**Table:**

Nonius KappaCCD diffractometer	5132 independent reflections
Radiation source: fine-focus sealed tube	4480 reflections with *I* > 2σ(*I*)
Graphite monochromator	*R*_int_ = 0.032
Detector resolution: 9 pixels mm^-1^	θ_max_ = 26.8°, θ_min_ = 1.9°
φ and ω scans with κ offset	*h* = −13→13
Absorption correction: multi-scan (*DENZO*/*SCALEPACK*; Otwinowski & Minor, 1997)	*k* = −14→14
*T*_min_ = 0.567, *T*_max_ = 0.708	*l* = −15→15
18801 measured reflections	

### Refinement

**Table:**

Refinement on *F*^2^	Primary atom site location: structure-invariant direct methods
Least-squares matrix: full	Secondary atom site location: difference Fourier map
*R*[*F*^2^ > 2σ(*F*^2^)] = 0.030	Hydrogen site location: inferred from neighbouring sites
*wR*(*F*^2^) = 0.082	H-atom parameters constrained
*S* = 1.02	*w* = 1/[σ^2^(*F*_o_^2^) + (0.0479*P*)^2^ + 0.3979*P*] where *P* = (*F*_o_^2^ + 2*F*_c_^2^)/3
5132 reflections	(Δ/σ)_max_ = 0.004
262 parameters	Δρ_max_ = 0.38 e Å^−^^3^
0 restraints	Δρ_min_ = −0.30 e Å^−^^3^

### Special details

**Table:**

Geometry. All esds (except the esd in the dihedral angle between two l.s. planes) are estimated using the full covariance matrix. The cell esds are taken into account individually in the estimation of esds in distances, angles and torsion angles; correlations between esds in cell parameters are only used when they are defined by crystal symmetry. An approximate (isotropic) treatment of cell esds is used for estimating esds involving l.s. planes.
Refinement. Refinement of F^2^ against ALL reflections. The weighted R-factor wR and goodness of fit S are based on F^2^, conventional R-factors R are based on F, with F set to zero for negative F^2^. The threshold expression of F^2^ > 2sigma(F^2^) is used only for calculating R-factors(gt) etc. and is not relevant to the choice of reflections for refinement. R-factors based on F^2^ are statistically about twice as large as those based on F, and R- factors based on ALL data will be even larger.

### Fractional atomic coordinates and isotropic or equivalent isotropic displacement parameters (Å^2^)

**Table:**

	*x*	*y*	*z*	*U*_iso_*/*U*_eq_	
Ge1	0.99264 (2)	0.176944 (19)	0.058635 (17)	0.02421 (8)	
O1	0.83353 (16)	0.07223 (14)	0.09872 (12)	0.0296 (3)	
O2	1.01794 (16)	0.34530 (13)	0.10758 (12)	0.0278 (3)	
N1	0.83055 (19)	0.16252 (16)	−0.11701 (14)	0.0263 (3)	
N2	0.4689 (2)	0.02892 (19)	−0.32794 (16)	0.0361 (4)	
C1	0.8348 (2)	−0.02668 (19)	0.15472 (18)	0.0267 (4)	
C2	0.9506 (2)	0.0077 (2)	0.27524 (18)	0.0275 (4)	
C3	0.9438 (3)	−0.0959 (2)	0.3307 (2)	0.0321 (4)	
H3	1.0220	−0.0722	0.4127	0.039*	
C4	0.8262 (3)	−0.2324 (2)	0.2697 (2)	0.0363 (5)	
C5	0.7122 (3)	−0.2634 (2)	0.1524 (2)	0.0377 (5)	
H5	0.6303	−0.3564	0.1098	0.045*	
C6	0.7121 (2)	−0.1637 (2)	0.09329 (19)	0.0329 (4)	
C7	1.0795 (3)	0.1543 (2)	0.3472 (2)	0.0388 (5)	
H7A	1.0320	0.2064	0.3541	0.047*	
H7B	1.1414	0.1940	0.3051	0.047*	
H7C	1.1488	0.1580	0.4290	0.047*	
C8	0.8247 (4)	−0.3426 (3)	0.3322 (3)	0.0530 (7)	
H8C	0.7347	−0.4325	0.2758	0.064*	
H8B	0.8150	−0.3262	0.4053	0.064*	
H8A	0.9234	−0.3403	0.3559	0.064*	
C9	0.5795 (3)	−0.2027 (3)	−0.0325 (2)	0.0535 (7)	
H9A	0.6226	−0.1755	−0.0887	0.064*	
H9B	0.5246	−0.1562	−0.0303	0.064*	
H9C	0.5049	−0.3019	−0.0606	0.064*	
C10	1.1231 (2)	0.42748 (17)	0.22520 (17)	0.0243 (4)	
C11	1.0634 (2)	0.44853 (18)	0.29946 (18)	0.0265 (4)	
C12	1.1684 (3)	0.52993 (19)	0.41937 (19)	0.0322 (4)	
H12	1.1284	0.5453	0.4698	0.039*	
C13	1.3313 (3)	0.5899 (2)	0.46805 (19)	0.0335 (5)	
C14	1.3866 (2)	0.5700 (2)	0.3916 (2)	0.0349 (5)	
H14	1.4974	0.6112	0.4233	0.042*	
C15	1.2861 (2)	0.49171 (19)	0.26980 (18)	0.0292 (4)	
C16	0.8876 (2)	0.3819 (2)	0.2466 (2)	0.0370 (5)	
H16A	0.8365	0.2823	0.2199	0.044*	
H16B	0.8474	0.4135	0.1764	0.044*	
H16C	0.8640	0.4062	0.3089	0.044*	
C17	1.4443 (3)	0.6754 (3)	0.6003 (2)	0.0502 (6)	
H17A	1.4193	0.6194	0.6539	0.060*	
H17B	1.4334	0.7534	0.6149	0.060*	
H17C	1.5531	0.7088	0.6179	0.060*	
C18	1.3512 (3)	0.4816 (2)	0.1866 (2)	0.0432 (5)	
H18A	1.3172	0.5193	0.1196	0.052*	
H18B	1.3118	0.3854	0.1531	0.052*	
H18C	1.4670	0.5334	0.2330	0.052*	
C19	0.6950 (2)	0.1647 (2)	−0.11538 (18)	0.0329 (4)	
H19A	0.7388	0.2484	−0.0523	0.039*	
H19B	0.6390	0.0855	−0.0894	0.039*	
C20	0.5744 (3)	0.1608 (2)	−0.2342 (2)	0.0449 (6)	
H20A	0.5092	0.1876	−0.2161	0.054*	
H20B	0.6315	0.2300	−0.2678	0.054*	
C21	0.9229 (3)	0.2794 (2)	−0.1534 (2)	0.0417 (5)	
H21A	1.0080	0.2729	−0.1562	0.050*	
H21B	0.9685	0.3653	−0.0937	0.050*	
H21C	0.8531	0.2774	−0.2343	0.050*	
C22	0.7796 (3)	0.0334 (2)	−0.19849 (18)	0.0346 (5)	
H22A	0.7297	−0.0434	−0.1690	0.042*	
H22B	0.8720	0.0387	−0.1985	0.042*	
H22C	0.7036	0.0198	−0.2818	0.042*	
C23	0.3876 (4)	0.0448 (4)	−0.4447 (3)	0.1074 (16)	
H23C	0.3178	−0.0442	−0.5069	0.129*	
H23B	0.4652	0.1081	−0.4671	0.129*	
H23A	0.3245	0.0814	−0.4395	0.129*	
C24	0.3532 (3)	−0.0687 (3)	−0.2991 (3)	0.0546 (7)	
H24C	0.2847	−0.1557	−0.3639	0.066*	
H24B	0.2889	−0.0341	−0.2927	0.066*	
H24A	0.4070	−0.0828	−0.2211	0.066*	

### Atomic displacement parameters (Å^2^)

**Table:**

	*U*^11^	*U*^22^	*U*^33^	*U*^12^	*U*^13^	*U*^23^
Ge1	0.02419 (12)	0.02539 (11)	0.02330 (11)	0.01458 (9)	0.00996 (9)	0.00678 (8)
O1	0.0305 (7)	0.0311 (7)	0.0319 (7)	0.0181 (6)	0.0163 (6)	0.0151 (6)
O2	0.0294 (7)	0.0243 (6)	0.0247 (7)	0.0144 (6)	0.0091 (6)	0.0040 (5)
N1	0.0302 (9)	0.0277 (8)	0.0231 (8)	0.0178 (7)	0.0119 (7)	0.0068 (6)
N2	0.0301 (9)	0.0457 (10)	0.0261 (9)	0.0187 (8)	0.0097 (8)	0.0113 (8)
C1	0.0299 (10)	0.0290 (10)	0.0315 (10)	0.0181 (8)	0.0199 (9)	0.0126 (8)
C2	0.0300 (10)	0.0289 (10)	0.0310 (10)	0.0180 (8)	0.0178 (9)	0.0111 (8)
C3	0.0375 (11)	0.0380 (11)	0.0349 (11)	0.0257 (10)	0.0217 (10)	0.0169 (9)
C4	0.0493 (13)	0.0330 (11)	0.0500 (13)	0.0273 (10)	0.0360 (12)	0.0225 (10)
C5	0.0444 (13)	0.0257 (10)	0.0463 (13)	0.0144 (9)	0.0296 (11)	0.0100 (9)
C6	0.0334 (11)	0.0312 (10)	0.0342 (11)	0.0142 (9)	0.0199 (9)	0.0086 (9)
C7	0.0419 (12)	0.0315 (11)	0.0329 (11)	0.0183 (10)	0.0109 (10)	0.0110 (9)
C8	0.0727 (18)	0.0409 (13)	0.0668 (17)	0.0355 (13)	0.0435 (15)	0.0311 (13)
C9	0.0435 (14)	0.0415 (13)	0.0410 (14)	0.0059 (11)	0.0116 (12)	0.0086 (11)
C10	0.0285 (10)	0.0181 (8)	0.0244 (9)	0.0117 (8)	0.0117 (8)	0.0068 (7)
C11	0.0320 (10)	0.0199 (9)	0.0311 (10)	0.0148 (8)	0.0165 (9)	0.0102 (7)
C12	0.0446 (12)	0.0246 (9)	0.0314 (11)	0.0184 (9)	0.0215 (10)	0.0090 (8)
C13	0.0387 (12)	0.0227 (9)	0.0278 (10)	0.0128 (9)	0.0107 (9)	0.0061 (8)
C14	0.0272 (10)	0.0272 (10)	0.0382 (12)	0.0105 (9)	0.0106 (9)	0.0072 (9)
C15	0.0284 (10)	0.0228 (9)	0.0332 (11)	0.0114 (8)	0.0148 (9)	0.0077 (8)
C16	0.0331 (11)	0.0348 (11)	0.0446 (13)	0.0171 (9)	0.0216 (10)	0.0073 (9)
C17	0.0535 (15)	0.0405 (13)	0.0327 (12)	0.0163 (12)	0.0116 (11)	0.0026 (10)
C18	0.0324 (12)	0.0428 (13)	0.0477 (14)	0.0118 (10)	0.0241 (11)	0.0054 (10)
C19	0.0312 (11)	0.0368 (11)	0.0298 (11)	0.0238 (9)	0.0079 (9)	0.0017 (8)
C20	0.0374 (12)	0.0371 (12)	0.0489 (14)	0.0239 (11)	0.0063 (11)	0.0132 (10)
C21	0.0507 (14)	0.0427 (13)	0.0325 (11)	0.0236 (11)	0.0212 (11)	0.0196 (10)
C22	0.0416 (12)	0.0387 (11)	0.0250 (10)	0.0268 (10)	0.0113 (9)	0.0015 (8)
C23	0.067 (2)	0.103 (3)	0.0475 (19)	0.004 (2)	−0.0124 (16)	0.0391 (19)
C24	0.0393 (14)	0.0576 (16)	0.0522 (16)	0.0156 (12)	0.0221 (12)	0.0133 (13)

### Geometric parameters (Å, º)

**Table:**

Ge1—O1	1.8760 (13)	C11—C16	1.502 (3)
Ge1—O2	1.8674 (13)	C12—C13	1.394 (3)
Ge1—N1	2.1261 (16)	C12—H12	0.9500
O1—C1	1.368 (2)	C13—C14	1.379 (3)
O2—C10	1.368 (2)	C13—C17	1.515 (3)
N1—C21	1.483 (3)	C14—C15	1.392 (3)
N1—C22	1.484 (2)	C14—H14	0.9500
N1—C19	1.498 (2)	C15—C18	1.504 (3)
N2—C24	1.438 (3)	C16—H16A	0.9800
N2—C23	1.441 (3)	C16—H16B	0.9800
N2—C20	1.459 (3)	C16—H16C	0.9800
C1—C2	1.399 (3)	C17—H17A	0.9800
C1—C6	1.403 (3)	C17—H17B	0.9800
C2—C3	1.396 (3)	C17—H17C	0.9800
C2—C7	1.507 (3)	C18—H18A	0.9800
C3—C4	1.388 (3)	C18—H18B	0.9800
C3—H3	0.9500	C18—H18C	0.9800
C4—C5	1.372 (3)	C19—C20	1.514 (3)
C4—C8	1.520 (3)	C19—H19A	0.9900
C5—C6	1.395 (3)	C19—H19B	0.9900
C5—H5	0.9500	C20—H20A	0.9900
C6—C9	1.501 (3)	C20—H20B	0.9900
C7—H7A	0.9800	C21—H21A	0.9800
C7—H7B	0.9800	C21—H21B	0.9800
C7—H7C	0.9800	C21—H21C	0.9800
C8—H8C	0.9800	C22—H22A	0.9800
C8—H8B	0.9800	C22—H22B	0.9800
C8—H8A	0.9800	C22—H22C	0.9800
C9—H9A	0.9800	C23—H23C	0.9800
C9—H9B	0.9800	C23—H23B	0.9800
C9—H9C	0.9800	C23—H23A	0.9800
C10—C15	1.396 (3)	C24—H24C	0.9800
C10—C11	1.400 (3)	C24—H24B	0.9800
C11—C12	1.386 (3)	C24—H24A	0.9800
			
O2—Ge1—O1	97.06 (6)	C12—C13—C17	121.1 (2)
O2—Ge1—N1	84.95 (6)	C13—C14—C15	122.4 (2)
O1—Ge1—N1	93.73 (6)	C13—C14—H14	118.8
C1—O1—Ge1	121.49 (11)	C15—C14—H14	118.8
C10—O2—Ge1	120.51 (11)	C14—C15—C10	118.36 (18)
C21—N1—C22	108.99 (16)	C14—C15—C18	120.90 (19)
C21—N1—C19	111.75 (17)	C10—C15—C18	120.68 (18)
C22—N1—C19	112.80 (15)	C11—C16—H16A	109.5
C21—N1—Ge1	106.26 (13)	C11—C16—H16B	109.5
C22—N1—Ge1	104.98 (12)	H16A—C16—H16B	109.5
C19—N1—Ge1	111.64 (12)	C11—C16—H16C	109.5
C24—N2—C23	108.7 (2)	H16A—C16—H16C	109.5
C24—N2—C20	111.8 (2)	H16B—C16—H16C	109.5
C23—N2—C20	110.3 (2)	C13—C17—H17A	109.5
O1—C1—C2	120.98 (17)	C13—C17—H17B	109.5
O1—C1—C6	119.40 (18)	H17A—C17—H17B	109.5
C2—C1—C6	119.48 (18)	C13—C17—H17C	109.5
C3—C2—C1	119.14 (18)	H17A—C17—H17C	109.5
C3—C2—C7	119.50 (19)	H17B—C17—H17C	109.5
C1—C2—C7	121.35 (17)	C15—C18—H18A	109.5
C4—C3—C2	122.0 (2)	C15—C18—H18B	109.5
C4—C3—H3	119.0	H18A—C18—H18B	109.5
C2—C3—H3	119.0	C15—C18—H18C	109.5
C5—C4—C3	117.77 (19)	H18A—C18—H18C	109.5
C5—C4—C8	121.7 (2)	H18B—C18—H18C	109.5
C3—C4—C8	120.5 (2)	N1—C19—C20	116.77 (18)
C4—C5—C6	122.5 (2)	N1—C19—H19A	108.1
C4—C5—H5	118.7	C20—C19—H19A	108.1
C6—C5—H5	118.7	N1—C19—H19B	108.1
C5—C6—C1	119.0 (2)	C20—C19—H19B	108.1
C5—C6—C9	120.2 (2)	H19A—C19—H19B	107.3
C1—C6—C9	120.79 (19)	N2—C20—C19	115.27 (18)
C2—C7—H7A	109.5	N2—C20—H20A	108.5
C2—C7—H7B	109.5	C19—C20—H20A	108.5
H7A—C7—H7B	109.5	N2—C20—H20B	108.5
C2—C7—H7C	109.5	C19—C20—H20B	108.5
H7A—C7—H7C	109.5	H20A—C20—H20B	107.5
H7B—C7—H7C	109.5	N1—C21—H21A	109.5
C4—C8—H8C	109.5	N1—C21—H21B	109.5
C4—C8—H8B	109.5	H21A—C21—H21B	109.5
H8C—C8—H8B	109.5	N1—C21—H21C	109.5
C4—C8—H8A	109.5	H21A—C21—H21C	109.5
H8C—C8—H8A	109.5	H21B—C21—H21C	109.5
H8B—C8—H8A	109.5	N1—C22—H22A	109.5
C6—C9—H9A	109.5	N1—C22—H22B	109.5
C6—C9—H9B	109.5	H22A—C22—H22B	109.5
H9A—C9—H9B	109.5	N1—C22—H22C	109.5
C6—C9—H9C	109.5	H22A—C22—H22C	109.5
H9A—C9—H9C	109.5	H22B—C22—H22C	109.5
H9B—C9—H9C	109.5	N2—C23—H23C	109.5
O2—C10—C15	121.18 (17)	N2—C23—H23B	109.5
O2—C10—C11	118.29 (17)	H23C—C23—H23B	109.5
C15—C10—C11	120.52 (17)	N2—C23—H23A	109.5
C12—C11—C10	118.91 (18)	H23C—C23—H23A	109.5
C12—C11—C16	122.22 (18)	H23B—C23—H23A	109.5
C10—C11—C16	118.88 (17)	N2—C24—H24C	109.5
C11—C12—C13	121.75 (19)	N2—C24—H24B	109.5
C11—C12—H12	119.1	H24C—C24—H24B	109.5
C13—C12—H12	119.1	N2—C24—H24A	109.5
C14—C13—C12	117.89 (19)	H24C—C24—H24A	109.5
C14—C13—C17	121.0 (2)	H24B—C24—H24A	109.5
			
O2—Ge1—O1—C1	−137.81 (14)	O1—C1—C6—C9	−0.3 (3)
N1—Ge1—O1—C1	136.83 (14)	C2—C1—C6—C9	175.4 (2)
O1—Ge1—O2—C10	89.17 (13)	Ge1—O2—C10—C15	68.9 (2)
N1—Ge1—O2—C10	−177.69 (14)	Ge1—O2—C10—C11	−112.58 (16)
O2—Ge1—N1—C21	61.23 (13)	O2—C10—C11—C12	179.06 (16)
O1—Ge1—N1—C21	158.00 (13)	C15—C10—C11—C12	−2.4 (3)
O2—Ge1—N1—C22	176.63 (13)	O2—C10—C11—C16	−0.8 (3)
O1—Ge1—N1—C22	−86.60 (13)	C15—C10—C11—C16	177.69 (18)
O2—Ge1—N1—C19	−60.84 (13)	C10—C11—C12—C13	−0.7 (3)
O1—Ge1—N1—C19	35.93 (13)	C16—C11—C12—C13	179.16 (19)
Ge1—O1—C1—C2	68.1 (2)	C11—C12—C13—C14	2.3 (3)
Ge1—O1—C1—C6	−116.27 (17)	C11—C12—C13—C17	−178.28 (19)
O1—C1—C2—C3	177.34 (16)	C12—C13—C14—C15	−0.8 (3)
C6—C1—C2—C3	1.7 (3)	C17—C13—C14—C15	179.8 (2)
O1—C1—C2—C7	−1.0 (3)	C13—C14—C15—C10	−2.3 (3)
C6—C1—C2—C7	−176.58 (19)	C13—C14—C15—C18	175.0 (2)
C1—C2—C3—C4	0.5 (3)	O2—C10—C15—C14	−177.68 (17)
C7—C2—C3—C4	178.86 (19)	C11—C10—C15—C14	3.9 (3)
C2—C3—C4—C5	−1.7 (3)	O2—C10—C15—C18	5.0 (3)
C2—C3—C4—C8	178.71 (19)	C11—C10—C15—C18	−173.42 (19)
C3—C4—C5—C6	0.7 (3)	C21—N1—C19—C20	58.8 (2)
C8—C4—C5—C6	−179.7 (2)	C22—N1—C19—C20	−64.5 (2)
C4—C5—C6—C1	1.4 (3)	Ge1—N1—C19—C20	177.61 (14)
C4—C5—C6—C9	−176.7 (2)	C24—N2—C20—C19	73.2 (3)
O1—C1—C6—C5	−178.36 (17)	C23—N2—C20—C19	−165.8 (3)
C2—C1—C6—C5	−2.7 (3)	N1—C19—C20—N2	73.3 (3)
